# Influence of Fish Species and Wood Type on Polycyclic Aromatic Hydrocarbons Contamination in Smoked Fish Meat

**DOI:** 10.3390/foods13121790

**Published:** 2024-06-07

**Authors:** Raul-Lucian Savin, Daniela Ladoși, Ioan Ladoși, Tudor Păpuc, Anca Becze, Oana Cadar, Iulia Torök, Dorina Simedru, Ștefania Codruța Mariș, Aurelia Coroian

**Affiliations:** 1 Faculty of Animal Science and Biotechnologies, University of Agricultural Sciences and Veterinary Medicine, 3-5 Mănăștur Street, 400372 Cluj-Napoca, Romania; raul.savin@usamvcluj.ro (R.-L.S.);; 2 INCDO-INOE2000, Research Institute for Analytical Instrumentation, 67 Donath Street, 400293 Cluj-Napoca, Romaniaoana.cadar@icia.ro (O.C.); iulia.torok@icia.ro (I.T.);; 3 Department of Environment and Soil Sciences, University of Lleida, UDL, Av. Rovira Roure, 191, 25198 Leida, Spain

**Keywords:** Benzo(a)Piren, contamination, food safety, human health risk, PAHs

## Abstract

Despite the numerous sensory, organoleptic and nutritional qualities, fish meat may also contain some toxic compounds with negative effects on human health. Polycyclic aromatic hydrocarbons (PAHs) are a class of chemicals resulting from incomplete combustion, found at high levels in thermally processed foods, especially in smoked fish. This research studied the influence of wood type (beech, plum and oak) and fish species (rainbow trout, carp and Siberian sturgeon) on PAH contamination in hot smoked fish. Benzo(a)Piren, Σ4PAHs and Σ15PAHs were considered as main indicators of PAH contamination. All-PAHs was quantified in all samples, indicating a specific dynamic of values due to the influence of variables. Generally, BaP (benzo(a)pyrene) content in the samples ranged from 0.11 µg/kg to 8.63 µg/kg, Σ4PAHs from 0.70 µg/kg to 45.24 µg/kg and Σ15PAHs from 17.54 µg/kg to 450.47 µg/kg. Thus, plum wood promoted the highest levels of PAHs, followed by oak and beech. Carp and Siberian sturgeon presented the highest concentrations of PAHs. Some of these parameters had levels that exceeded the limits allowed by legislation via Commission Regulation (EU) No 835/2011. Results revealed BaP levels > 2 µg/kg when plum wood was used in rainbow trout (4.04 µg/kg), carp (4.47 µg/kg) and Siberian sturgeon (8.63 µg/kg). Moreover, the same trend was found for Σ4PAHs, which exceeded 12 µg/kg in rainbow trout (17.57 µg/kg), carp (45.24 µg/kg) and Siberian sturgeon (44.97 µg/kg).

## 1. Introduction

Fish meat is consumed worldwide for its nutritional and organoleptic qualities [1]. Beyond these basic qualities, many authors consider fish meat a functional food, as it provides food constituents with high biological value, which can improve human health [2,3]. Fish meat is consumed as a quality food in many ways, from raw to various fish products, as a result of certain processing technologies or domestic cooking practices. Along with all these beneficial nutrients, fish meat can also contain some notable contaminants, mainly due to bioaccumulation [4] or contamination via food processing methods [5]. Among contaminants of chemical origin, polycyclic aromatic hydrocarbons (PAHs) are of interest to both consumers and researchers for the possible or demonstrated carcinogenic, mutagenic and tetragenic effects [6]. PAHs are a complex group of hundreds of different organic compounds consisting mainly of carbon and hydrogen, with multiple fused aromatic rings in various structural configurations [7,8,9]. Some general physical and chemical properties of PAHs may promote the occurrence in fish meat. Properties such as high melting and boiling points, low vapor pressure, high lipophilic, light sensitivity, hydrophobicity, conductivity, emittability and resistance to corrosion [10,11] may influence biological activity and metabolic activation, bioaccumulation and toxicity [12,13]. All these characteristics largely depend on PAHs’ molecular weight. PAHs can be classified as having a high molecular weight (HMW) with four or more aromatic rings fused together or low molecular weight (LMV) with less than four aromatic rings fused together [8]. PAHs are formed by pyrolysis and pyrosynthesis processes, promoted by saturated hydrocarbon under oxygen-deficient conditions [14].

Generally, there are two sources of PAHs in fish meat: bioaccumulation ia environmental sources [15] and contamination via food processing methods. Most PAHs in fish meat are generated directly during heat treatments such as roasting [16,17], barbequing [18,19], grilling [20,21], frying [22,23] and smoking [24,25]. Numerous comparative studies have concluded that smoked fish contains the highest amounts of PAHs [26,27,28]. Smoking is a chemical process used as a method for food preparation, in which smoke is produced by combustion or incomplete combustion. Several aspects are pursued by the smoking process, such as improving the organoleptic quality and preserving and extending the shelf life of food. Wood smoke is a heterogeneous mixture of gaseous and particulate matter [29] containing a complex composition of chemical compounds: acids, alcohols, carbonyls, esters, furans, lactones and phenols [30]. From ancient methods of smoking, such as directly hanging the food over a firebox, new methods of smoking have been improved. Thus, nowadays, hot smoking, cold smoking and liquid smoking with different smoke generators are known and largely used. 

According to Codex Alimentarius Commission CAC/RCP 68/2009 [31], there are some major variables of the smoking process that may contribute to PAH contamination, including the smoking method, fuel type, temperature, duration, distance between food and smoke generator and type of food. Many studies have been carried out around these variables, and the reported data vary widely because of differences in the procedures used for the evaluation of PAHs, meat composition and smoking methods [27]. Some authors have extensively studied the subject via review articles including relevant aspects or studied specific aspects individually or in comparison [11,27]. 

Generally, PAH indicators in food are evaluated in terms of the sum of PAHs. While the United States Environmental Protection Agency (USEPA) suggests the set of ∑16PAHs (benz(a)anthracene, benzo(a)pyrene, benzo(b)fluoranthene, benzo(k)fluoranthene, chrysene, dibenz(a,h)anthracene, indeno(1,2,3-cd)pyrene, acenaphthene, acenaphthylene, anthracene, benzo(ghi)perylene, fluoranthene, fluorene, naphthalene, phenanthrene and pyrene) for evaluation, the European Food Safety Agency (EFSA) suggests the set of ∑4PAHs (benzo[a]pyrene, chrysene, benz[a]anthracene and benzo[b]fluoranthene) or ∑8PAHs (benzo[a]pyrene, benz[a]anthracene, benzo[b]fluoranthene, benzo[k]fluoranthene, benzo[ghi]perylene, chrysene, dibenz[a,h]anthracene and indeno [1,2,3-cd]pyrene) for evaluation. At the same time, the approach with only one compound is largely accepted, as can be seen in Commission Regulation (EU) No 835/2011, wherein benzo[a]pyrene (BaP) is considered the most relevant among PAHs, alongside benz[a]anthracene, benzo[b]fluoranthene and chrysene. The European Commission sets the maximum limits for these indicators for smoked fish: for BaP, the limit is 2 µg/kg, and for ∑4PAHs, the limit is 12 µg/kg [32]. All these standards and regulations are established for the food safety of consumers to protect them from the negative health effects of PAHs.

Although research on PAHs in food already has a long history, the reported data varies widely. Smoked fish is a specific food product due to the local materials used. Generally, there are many fish species and types of wood used with different physico-chemical and biological characteristics. There are countless variations for each smoking method in each of its steps. For example, the vegetal material used can be singular (beech) or mixed (beech an cherry), with stifling carried out by other vegetal materials (fir and nettle) [33]. Further studies are needed due to the complexity of the concept, especially as PAHs and BaP levels are legislated in the EU, and in many cases, the threshold is exceeded [34].

This study aimed to determine quantitative and qualitative levels of PAHs in fish meat depending on the fish species and the type of wood used for smoking. Thus, the objective of this research was to determine whether the direct hot smoking of 3 species of fish (Siberian sturgeon, *Acipenser baerii*; carp, *Cyprinus carpio* and rainbow trout, *Oncorhynchus mykiss*) with three different wood species (oak, *Quercus robur*; beech, *Fagus sylvatica* and plum, *Prunus domestica*) influences PAH content accumulation under the same smoking method based on species and wood type. Among the factors that have a major influence on the PAH content of smoked fish, we considered the analysis of the interaction between the fish species and the type of wood used as a novel element. The overall goal is to contribute to the improvement in PAH reduction methods in smoked fish products in order to control their safety.

## 2. Materials and Methods

### 2.1. Biological Material

The fish species were selected based on accessibility and the chemical composition of meat. The fish selected for smoking must be as fresh as possible, so we focused on nearby fisheries. Thus, we chose to use Siberian sturgeon, common carp and rainbow trout. All species were farmed in a semi-intensive system, using granulated feed from established international brands specific to the nutritional requirements of each species. Fish were bought, eviscerated and portioned as medallions for the intended purpose, namely smoking. More specifically, the number of samples consisted of 135 fish medallions, 45 per species, 15 per repetition, of 200 g each, which were stored cold at the recommended temperature of 0–2 °C for a maximum of 4 h until preparation for smoking. In terms of bioethical principles, no authorization or ethical approval was necessary from the affiliated institutions because no live biological material was used in the experiment. Fisheries are authorized and specialized in eviscerating fish and ensuring quality and safety standards, as they farm fish for meat production.

### 2.2. Smoking Type, Materials and Experimental Protocol

The three wood species used as fuel material for smoking were selected according to the following considerations: beech is locally available and very often used as a single hard wood material for its mild flavors; plum is used only as secondary material to enhance sweet flavor and a red-gold color of meat; oak is a versatile hard wood used worldwide in barbecuing, grilling and even smoking for its strong flavors. Wood materials were bought from specific suppliers who guaranteed that wood was free from any chemicals that could have been used in wood processing. The wood chunks were approximately the same size (35 × 5 × 5 cm) and had approximately the same moisture (10 ± 2%).

A commercial smokehouse was used, made from stainless steel, which works on the principle of continuous direct hot smoke. It has two chambers: one chamber where meat is hung on hangers under the smoke stream and the second where smoke is generated. The food chamber is equipped with a thermometer and hygrometer for controlling these parameters. Between two batches of fish for smoking, the smokehouse was cleaned, and the combustion material and burnt material were removed.

The small-scale smoking technological flow included the following steps: receiving fish meat samples, rinsing with water (to ensure hygiene standards), draining (until the excess water has been removed, about 30 min), salting (dry salting for 12 h, under refrigeration temperature 2 ± 1 °C) and surface cold drying (for 6 h, under refrigeration temperature 2 ± 1 °C).

The smoking process started with preheating. The fire was started using sawdust and splinters from the same type of wood as the combustion material, using a lighter. When the temperature reached 80 °C, meat hangers were introduced. There were 15 hangers in each smoking procedure (5 per species), arranged randomly. At this temperature ±10 °C, the fish meat was smoked for 3 h, after which, for an hour, the temperature gradually decreased to the ambient temperature of 20 °C. The humidity lightly fluctuated between 45 and 50%. There were 3 such repetitions for each wood species. When the meat was completely cooled, it was transferred for sampling. The meat from each species of fish was manually separated from the skin, bones and fins. The whole amount of meat from each medallion was cut into small pieces using a clean ceramic knife, homogenized and chopped with an electric meat grinder. Thus, a number of five samples resulted from each species, in each repetition, for each wood type for analysis. The packaging was performed in polyethylene containers and stored at a temperature of −25 °C until further analyses were performed.

### 2.3. Fish Proximate Composition

Prior to the smoking technological flow, fish meat was randomly sampled for the proximate composition analysis, totaling 450 g from every fish species. The sampling was carried out similar to the sampling of smoked meat. This amount was cut into small pieces using a clean ceramic knife, homogenized and chopped with an electric meat grinder. Homogenized samples were stored at a temperature of −25 °C until further analytical chemical investigations. For proximate composition evaluation, AOAC standard protocols were used, as follows: lipids, moisture and protein via AOAC 2007.04-2007 method [35] and total minerals via AOAC 920.153-1920 [36].

The dry matter (DM) was determined as the difference between 100 g of sample and the moisture contents:

DM (g/100 g) = 100 g − moisture (g/100 g)

The total organic matter (TOM) was calculated as the difference between DM and total mineral (TM) contents:

TOM (g/100 g) = DM (g/100 g) − TM (g/100 g)

### 2.4. PAHs Analysis

The PAHs analysis method is based on the use of high-performance liquid chromatography (HPLC) with fluorescence detection after solid–liquid extraction for the determination of 15 PAHs: naphthalene, acenaphthene, fluorene, phenanthrene, anthracene, fluoranthene, pyrene, benzo(a)anthracene, chrysene, benzo(a)pyrene, benzo(b) fluoranthene, benzo(k)fluoranthene, benzo(gy)pyrylene, dibenzo(a,h)anthracene and indeno(1,2,3-cd)pyrene.

The analysis was carried out according to the following steps: for extraction, 10 g of the sample was selected and homogenized in an electric blender; 50 mL of 0.4 M KOH solution in ethanol and water (9:1) was added. Saponification was conducted using an ultrasonic bath for 30 min at 60 °C. After filtering on filter paper, the obtained product was extracted twice with 15 mL of cyclohexane. The supernatant was purified on a Florisil column, after which it was evaporated to dryness under a flow of nitrogen, and finally, it was reduced with 1 mL of acetonitrile. Before injection, the samples were filtered on 0.45 µm cartridges.

The following calculation formula was used to express the results:

Concentration [μg/kg] = (C × D × R)/Mp × 1000

where C—HPLC ng/mL amount; D—the dilution factor; Mp—sample weight in g; R—recovery%

### 2.5. Statistical Analysis

All data were analyzed with the Graph Pad Prism 9.4.1 software (GraphPad Inc., Palo Alto, CA, USA) in order to achieve the main descriptive statistics values. The normal distribution of all the data was verified using the Shapiro–Wilk test. When necessary, in order to fulfill the assumption of normality, data were log-transformed prior to analysis. We used ANOVA one-way, followed by the Tukey post hoc test when 3 data groups were compared, or the unpaired 2-tailed *t* test when just 2 data groups were compared. Comparisons were carried out among fish species and wood types to determine if particularities of fish species or wood species can influence PAH contamination in the final product. Moreover, an average of PAH accumulation was examined using data from all parameters measured with an ANOVA model (JMP version 10, SAS Institute, Cary, NC, USA) that included terms for fish species (rainbow trout, carp and Siberian sturgeon), wood species (beech wood, plum wood and oak wood) and their interaction (fish species ×wood species). Significant differences among treatment means were further examined using Tukey’s multiple range test at the 0.001 probability level.

## 3. Results

The chemical composition of the meat prior to smoking is presented in Table 1. The values are specific for semi-intensive growth systems, with higher values observed for lipids and lower values for proteins. Thus, trout had the highest crude protein value (18.62 g/100 g), followed by sturgeon (16.11 g/100 g) and carp (12.20 g/100 g). Fat content was highest in carp, reaching 17.44 g/100 g, compared to the low content in trout, 4.75 g/100 g. In general, moisture content is inversely proportional to the lipid content, this fact being observed in carp.

The first smoking variable studied was the influence of wood type on the PAH content. Table 2 shows the mean and standard deviation of PAH concentrations for each fish species under the influence of wood type, resulting from the ANOVA test analysis. As can be seen in Table 2, the *p*-value is less than 0.0001 for all samples, which implies high statistical significance. Due to this, Duncan’s post hoc test was applied to make a comparison of the samples. Overall, plum wood produced the highest content of PAHs, followed by oak wood and beech wood. These results show significant differences, demonstrating that the type of wood can influence the PAH content in smoked fish.

Interestingly, in the case of beech, the highest amounts of the majority of PAHs can be observed in Siberian sturgeon (BaP = 0.28 µg/kg, Σ15PAHs = 26.83 µg/kg), in comparison with carp (BaP = 0.14 µg/kg, Σ15PAHs = 21.52 µg/kg) and rainbow trout (BaP = 0.11 µg/kg, Σ15PAHs = 17.54 µg/kg), with the exception of chrysene and naphthalene. These two compounds substantially change the value of the Σ4PAHs indicator, as carp (Σ4PAHs = 2.06 µg/kg) and Siberian sturgeon (Σ4PAHs = 2.05 µg/kg) show similar values.

Regarding the influence on the chemical composition of fish smoked with plum, the values are surprising. The Σ15PAHs indicator had a similar value in rainbow trout (362.17 µg/kg) and carp (361.35 µg/kg), but the Σ4PAHs indicator had a significantly higher concentration in carp (45.24 µg/kg) than in rainbow trout (*p* < 0.0001). In addition, naphthalene had the highest amounts in trout (34.04 µg/kg) and carp (32.25 µg/kg), with a significantly lower (*p* < 0.0001) concentration in Siberian sturgeon (19.78 µg/kg).

For oak, the Σ4PAHs indicator is substantially higher for carp (21.55 µg/kg) (*p* < 0.0001). The same happens for BaP (1.69 µg/kg). It can be said that the highest amount of Σ15PAHs (104.94 µg/kg) in Siberian was due to naphthalene, fluorene, phenanthrene, anthracene and fluoranthene at high concentrations.

The average of PAHs in Siberian sturgeon was significantly larger compared to rainbow trout and crap, but not for naphthalene, crysene, benzo(b)fluoranthene, benzo(k)fluoranthene, benzo(a)pyrene, benzo(gy)pyrylene (Table 3). For all the parameters, the average of PAHs was significantly larger for the beech wood than the other two types of wood. Also, the interaction between the two factors was significant for all the parameters measured.

## 4. Discussion

The results showed a great variability according to fish species and type of wood. The all-PAHs indicator was quantified in all samples, indicating a specific dynamic of values. The main indicators of PAH accumulation were BaP, Σ4PAHs and Σ15PAHs, but also individual PAHs. Some of these parameters indicated levels that exceed the limits allowed by legislation via Commission Regulation (EU) No 835/2011; specifically, for smoked fish, BaP should not exceed 2 µg/kg, and Σ4PAHs should not exceed 12 µg/kg. The objective of this research was to determine if the type of wood and fish species can influence the accumulation of PAHs as a result of the smoking process.

### 4.1. Fish Species Influence on PAH Contamination in Smoked Fish

Regarding fish species, several aspects are worth discussing. On one hand, PAHs have a pronounced lipophilic character [37]. Thus, lipids are the main accumulation matrix for PAHs [38]. On the other hand, some authors may attribute higher PAH contamination to fat dripping into fire [39]. However, Essumang et al. [40] specified that fat molecules are transformed under thermal action at a temperature of 200 °C and favor PAH production at >500 °C.

The main reason we have chosen the common carp is for its high lipid content [41]. Results confirm the highest value for lipids (17.44 g/100 g) in common carp, followed by Siberian sturgeon (5.98 g/100 g) and rainbow trout (4.75 g/100 g). This chemical composition is to be expected when the fish are farmed in a semi-intensive system, with other authors indicating similar values [42]. The structure of the skin and muscle tissue is another specific feature of the fish species that can influence the migration and deposition of PAHs [43]. Sometimes, the skin or membrane cover of the food product can be a barrier more or less permissive in the migration of particles [38].

Considering these aspects and comparing the PAH values, it can be observed that the species of fish significantly influence PAH contamination. Generally, *p*-values indicate high statistical significance for all samples. In comparison, for BaP, the highest levels were found in Siberian sturgeon when beech (0.28 µg/kg) and plum (8.63 µg/kg) wood were used. Carp had the highest level when oak wood was used (1.69 µg/kg) and rainbow trout had the lowest levels for all three types of wood. In the case of Σ4PAHs, the values were slightly different because carp had the highest levels, followed by Siberian sturgeon and rainbow trout. Interestingly, after quantifying all the values of PAHs via Σ15PAHs, it can be clearly observed that the Siberian sturgeon had much higher values than the other species. The higher levels of PAHs in these samples may be partly attributed to the high lipid content of carp and the permissive structure of the sturgeon’s skin. In addition, the lower values in rainbow trout may be due to the scaly skin, which may be a natural barrier to PAH migration [38].

In a comparative study, the authors found that PAH levels in smoked fish could be attributed to the differences in fat and moisture contents, as they were higher in smoked mackerel than in sardine, cigar minnows or tuna [40]. Other studies, not necessarily on fish meat, demonstrated that the fat content and membrane of the smoked product had a major influence on PAH contamination [25,44,45].

### 4.2. Influence of Fuel Type on PAH Contamination in the Smoking Process

The type of fuel is one of the main factors that promote high levels of PAHs in smoked products [46]. When wood is used as fuel, it can influence the processes of pyrolysis and pyrosynthesis during the smoking of a product. The wood components involved in the production of smoke are cellulose, hemicellulose and lignin [47]. Of these chemical constituents, lignin content can be correlated with PAH production as it is the main macromolecular compound of wood, with complex aromatic characteristics and containing benzen rings [48]. Thus, lignin can be considered an aromatic precursor of PAH) [49].

The wood types used for smoking indicate high statistical significance (*p*-value < 0.0001). In other studies, it was determined that plum wood has the highest amount of lignin, 32.4–32.6% of the three utilized species [50,51]. For oak and beech wood, the data reported is largely variable, generally being in the range of 17.5–22.43% [51,52,53].

Samples analyzed for all PAH indicators (BaP, Σ4PAHs, Σ15PAHs) show the highest values when plum wood was used in comparison with beech wood and oak wood. There is only one exception for chrysene, which was higher in rainbow trout smoked with oak wood (0.36 µg/kg) than in rainbow trout smoked with plum wood (0.30 µg/kg). The lowest levels of PAH indicators occurred when beech wood was used, but when comparing individual PAHs, the levels of naphthalene in carp smoked with beech wood (4.44 µg/kg) were higher than in carp smoked with oak wood (4.40 µg/kg). In addition, for dibenzo(a,h)anthracene, levels were higher in Siberian sturgeon smoked with beech (0.11 µg/kg) than in Siberian sturgeon smoked with oak wood (0.10 µg/kg). These results certainly demonstrate that plum wood leads to major PAH deposition in fish meat.

Similar trends have been reported in several studies for various wood types. In a similar study [52], the authors concluded that meat smoked with spruce had much higher concentrations of PAHs (Σ15PAHs = 470.91 µg/kg) in comparison with meat smoked with apple wood, which had the lowest concentrations (Σ15PAHs = 47.94 µg/kg), with alder, maple, hazel, plum and aspen wood smoked meat (75.30 µg/kg–343.66 µg/kg) presenting intermediary values [54]. Surprisingly, values are relatively similar to the values found in our study, 324.67 µg/kg in smoked pork meat compared with 362.17 µg/kg in smoked rainbow trout meat and 361.35 µg/kg in smoked carp meat were observed when plum wood was utilized.

## 5. Conclusions

The results of this study improve the existing database on polycyclic aromatic hydrocarbons in smoked fish, providing clear differences in polycyclic aromatic hydrocarbons according to the species of fish and type of wood used. Polycyclic aromatic hydrocarbons influence the safety and quality of smoked fish, their levels being of interest to consumers and producers alike.

Our results show that the variables of wood type and fish species influence the levels of PAHs during the smoking process. Polycyclic aromatic hydrocarbons’ highest values appeared when carp and Siberian sturgeon were smoked. In this study, plum wood produced the highest levels of polycyclic aromatic hydrocarbons in the three fish species studied, thus making it a less suitable fuel for smoke generation. All values exceeded the limits allowed by the legislation when plum wood was used; is worrisome, especially as plum wood is used in traditional hot smoking.

Our recommendation is the use of beech wood for smoking rainbow trout and avoiding plum wood for smoking fish when beech or oak is available. Future studies should focus on the formation mechanisms, molecular properties and migration pathways of polycyclic aromatic hydrocarbons for these fish species of interest smoked with the common types of wood presented in the current study. Another direction of study that can be pursued is the determination of polycyclic aromatic hydrocarbons for the same fish species and wood types but processed by cold smoking to observe possible differences in polycyclic aromatic hydrocarbons accumulation.

## Figures and Tables

**Table 1:** Proximate composition of fish meat prior to smoking (g/100 g).

Proximate Compounds	Fish Species		
	Rainbow Trout (g/100 g)	Carp (g/100 g)	Siberian Sturgeon (g/100 g)
**Moisture**	75.22	68.65	76.82
**Dry matter**	24.78	31.35	23.18
**Total minerals**	1.33	1.6	1.03
**Organic matters**	23.45	29.75	22.15
**Total proteins**	18.62	12.20	16.11
**Total lipids**	4.75	17.44	5.98

**Table 2:** Analysis of total PAH accumulation in fish meat, under the influence of wood species.

PAH	Rainbow Trout			Carp			Siberian Sturgeon		
	Beech Wood	Plum Wood	Oak Wood	Beech Wood	Plum Wood	Oak Wood	Beech Wood	Plum Wood	Oak Wood
**Naph**	3.81 ± 0.17 ^e^	34.04 ± 0.93 ^a^	5.85 ± 0.69 ^d^	4.44 ± 0.57 ^cd^	32.25 ± 0.87 ^a^	4.40 ± 0.28 ^cd^	2.72 ± 0.71 ^e^	19.78 ± 0.33 ^b^	11.85 ± 0.74 ^c^
**Ace**	0.06 ± 0.01 ^c^	0.48 ± 0.09 ^ab^	0.08 ± 0.02 ^c^	0.07 ± 0.02 ^c^	0.42 ± 0.15 ^b^	0.12 ± 0.07 ^c^	0.11 ± 0.03 ^c^	0.68 ± 0.11 ^a^	0.45 ± 0.03 ^b^
**Flu**	1.27 ± 0.11 ^d^	55.17 ± 2.05 ^a^	7.29 ± 0.31 ^c^	1.57 ± 0.29 ^d^	55.45 ± 1.77 ^a^	8.63 ± 1.40 ^c^	1.60 ± 0.26 ^d^	56.55 ± 0.65 ^a^	13.24 ± 0.65 ^b^
**Phen**	4.60 ± 0.04 ^e^	74.18 ± 1.25 ^b^	12.52 ± 0.47 ^d^	5.67 ± 0.63 ^e^	74.21 ± 1.98 ^b^	14.04 ± 0.36 ^d^	6.53 ± 0.25 ^e^	85.59 ± 1.19 ^a^	26.94 ± 1.18 ^c^
**Ant**	1.36 ± 0.08 ^f^	24.92 ± 3.39 ^c^	3.53 ± 0.23 ^e^	1.79 ± 0.34 ^f^	26.66 ± 0.51 ^b^	3.62 ± 0.53 ^e^	1.99 ± 0.21 ^ef^	29.67 ± 1.53 ^a^	7.51 ± 0.65 ^d^
**Flt**	4.39 ± 0.04 ^f^	96.30 ± 2.58 ^b^	12.75 ± 1.25 ^e^	5.11 ± 0.54 ^f^	91.13 ± 1.77 ^c^	11.10 ± 0.42 ^e^	6.39 ± 0.75 ^f^	113.88 ± 2.59 ^a^	19.38 ± 0.60 ^d^
**Pyr**	2.45 ± 0.07 ^fg^	54.46 ± 1.95 ^b^	7.53 ± 0.58 ^e^	1.47 ± 0.41 ^g^	26.55 ± 0.61 ^c^	15.99 ± 1.06 ^d^	4.84 ± 0.13 ^ef^	87.23 ± 1.74 ^a^	15.17 ± 0.82 ^d^
**B(a)A**	0.50 ± 0.04 ^e^	12.47 ± 0.77 ^c^	0.54 ± 0.11 ^e^	0.95 ± 0.51 ^e^	19.06 ± 1.05 ^b^	4.56 ± 0.44 ^d^	1.07 ± 0.38 ^e^	20.48 ± 0.56 ^a^	3.54 ± 0.46 ^d^
**Cry**	0.01 ± 0.00 ^d^	0.30 ± 0.03 ^d^	0.36 ± 0.09 ^d^	0.85 ± 0.41 ^d^	18.80 ± 1.46 ^a^	10.43 ± 0.87 ^b^	0.51 ± 0.07 ^d^	11.42 ± 0.75 ^b^	2.80 ± 0.54 ^c^
**B(b)F**	0.02 ± 0.01 ^c^	0.76 ± 0.11 ^c^	0.53 ± 0.43 ^c^	0.12 ± 0.02 ^c^	2.92 ± 0.95 ^b^	4.87 ± 0.22 ^b^	0.19 ± 0.09 ^c^	4.44 ± 0.58 ^a^	0.93 ± 0.07 ^c^
**B(k)F**	0.06 ± 0.02 ^d^	2.12 ± 0.51 ^b^	1.75 ± 0.22 ^bc^	0.16 ± 0.10 ^d^	4.60 ± 0.98 ^a^	4.30 ± 0.36 ^a^	0.15 ± 0.08 ^d^	4.24 ± 0.61 ^a^	0.79 ± 0.14 ^cd^
**B(a)P**	0.11 ± 0.02 ^e^	4.04 ± 0.42 ^b^	0.47 ± 0.07 ^de^	0.14 ± 0.06 ^e^	4.47 ± 0.95 ^b^	1.69 ± 0.22 ^c^	0.28 ± 0.05 ^de^	8.63 ± 1.01 ^a^	0.68 ± 0.04 ^d^
**D(a,h)A**	0.03 ± 0.01 ^b^	0.59 ± 0.21 ^b^	0.07 ± 0.03 ^b^	0.04 ± 0.03 ^b^	0.58 ± 0.20 ^b^	0.16 ± 0.01 ^b^	0.11 ± 0.04 ^b^	1.46 ± 0.51 ^a^	0.10 ± 0.04 ^b^
**B(ghi)P**	0.09 ± 0.06 ^f^	2.20 ± 0.34 ^c^	1.34 ± 0.13 ^cd^	0.20 ± 0.08 ^ef^	4.09 ± 0.55 ^b^	3.69 ± 0.44 ^b^	0.26 ± 0.04 ^ef^	5.14 ± 0.49 ^a^	1.06 ± 0.33 ^de^
**I(1,2,3-cd)P**	0.05 ± 0.06 ^c^	0.05 ± 0.02 ^c^	0.05 ± 0.01 ^c^	0.05 ± 0.02 ^c^	0.05 ± 0.02 ^c^	0.05 ± 0.02 ^c^	0.05 ± 0.02 ^c^	0.20 ± 0.12 ^b^	0.42 ± 0.09 ^a^
**Σ4PAHs**	0.64 ± 0.04 ^b^	17.57 ± 0.12 ^e^	1.90 ± 0.05 ^a^	2.06 ± 0.43 ^a^	45.24 ± 0.39 ^cd^	21.55 ± 0.07 ^d^	2.05 ± 0.24 ^a^	44.97 ± 0.02 ^cd^	7.95 ± 0.44 ^b^
**Σ15PAHs**	17.54 ± 0.49 ^f^	362.17 ± 11.34 ^b^	54.69 ± 2.86 ^e^	21.52 ± 2.04 ^f^	361.35 ± 8.46 ^b^	87.66 ± 1.57 ^d^	26.83 ± 1.26 ^f^	450.47 ± 6.94 ^a^	104.94 ± 2.93 ^c^

Note: different superscripts show significant differences for *p* < 0.0001; Naph—naphthalene; Ace—acenaphthene; Flu—fluorene; Phen—phenanthrene; Ant—anthracene; Flt—fluoranthene; Pyr—pyrene; B(a)A—benzo(a)anthracene; Cry—chrysene; B(b)F—benzo(b) fluoranthene; B(k)F—ben-zo(k)fluoranthene; B(a)P—benzo(a)pyrene; D(a,h)A—dibenzo(a,h)anthracene; B(ghi)P—benzo(gy)pyrylene; I(1,2,3-cd)P—in-deno(1,2,3-cd)pyrene; Σ4PAHs—sum of benzo[a]pyrene, chrysene, benz[a]anthracene and benzo[b]fluoranthene values.

**Table 3:** Average of PAHs and standard deviation in fish meat per studied factor (wood species, fish species and their interaction).

Factor	Naph	Ace	Flu	Phen	Ant	Flt	Pyr	B(a)A	Cry	B(b)F	B(k)F	B(a)P	D(a,h)A	B(ghi)P	I (1,2,3-cd)P	Σ15 PAHs
**Fish species**																
Rainbow trout	14.57 ± 14.64 ^a^	0.22 ± 0.21 ^b^	21.25 ± 5.60 ^b^	30.45 ± 7.81 ^b^	9.94 ± 4.29 ^c^	37.81 ± 4.04 ^b^	21.48 ± 4.87 ^b^	4.51 ± 1.99 ^b^	0.25 ± 0.17 ^c^	0.44 ± 0.39 ^c^	1.32 ± 0.94 ^b^	1.55 ± 0.90 ^c^	0.24 ± 0.11 ^b^	1.21 ± 0.94 ^c^	0.048 ± 0.01 ^b^	144.80 ± 14.22 ^c^
Carp	13.70 ± 13.93 ^b^	0.21 ± 0.18 ^b^	21.87 ± 5.38 ^b^	31.31 ± 8.40 ^b^	10.69 ± 5.02 ^b^	35.78 ± 4.61 ^c^	14.67 ± 1.92 ^c^	8.20 ± 3.33 ^a^	10.03 ± 3.83 ^a^	2.64 ± 0.89 ^a^	3.03 ± 1.21 ^a^	2.10 ± 0.90 ^b^	0.27 ± 0.13 ^b^	2.66 ± 1.28 ^a^	0.045 ± 0.01 ^b^	156.84 ± 16.10 ^b^
Siberian sturgeon	11.46 ± 7.41 ^c^	0.42 ± 0.25 ^a^	23.80 ± 5.08 ^a^	39.69 ± 5.55 ^a^	13.06 ± 6.09 ^a^	46.56 ± 5.82 ^a^	35.75 ± 8.88 ^a^	8.70 ± 3.66 ^a^	4.91 ± 1.99 ^b^	1.86 ± 0.99 ^b^	1.73 ± 0.93 ^b^	3.20 ± 1.80 ^a^	0.26 ± 0.08 ^a^	2.15 ± 1.29 ^b^	0.220 ± 0.18 ^a^	194.06 ± 15.26 ^a^
**Wood species**																
Beech wood	3.67 ± 0.88 ^c^	0.09 ± 0.03 ^c^	1.50 ± 0.25 ^c^	5.60 ± 0.90 ^c^	1.72 ± 0.33 ^c^	5.31 ± 0.99 ^c^	2.92 ± 1.51 ^c^	0.84 ± 0.41 ^c^	0.46 ± 0.42 ^c^	0.12 ± 0.08 ^c^	3.66 ± 0.08 ^c^	0.18 ± 0.10 ^c^	0.07 ± 0.05 ^b^	0.18 ± 0.09 ^c^	0.047 ± 0.01 ^b^	21.95 ± 4.22 ^c^
Plum wood	28.70 ± 6.76 ^a^	0.53 ± 0.16 ^a^	55.72 ± 1.53 ^a^	77.99 ± 5.90 ^a^	27.09 ± 2.20 ^a^	100.44 ± 10.55 ^a^	56.08 ± 6.35 ^a^	17.68 ± 4.10 ^a^	10.18 ± 2.10 ^a^	2.71 ± 1.07 ^a^	3.66 ± 1.32 ^a^	5.72 ± 1.21 ^a^	0.88 ± 0.53 ^a^	3.81 ± 1.35 ^b^	0.096 ± 0.10 ^b^	391.32 ± 45.02 ^a^
Oak wood	7.37 ± 0.89 ^b^	0.22 ± 0.05 ^b^	9.71 ± 0.99 ^b^	17.83 ± 0.85 ^b^	4.90 ± 0.32 ^b^	14.41 ± 0.91 ^b^	12.90 ± 4.28 ^b^	2.88 ± 1.02 ^b^	4.53 ± 1.05 ^b^	2.11 ± 1.19 ^b^	2.30 ± 0.88 ^b^	0.95 ± 0.62 ^b^	0.12 ± 0.05 ^b^	20.30 ± 4.18 ^a^	0.170 ± 0.01 ^a^	82.43 ± 16.62 ^b^
Fish species	<0.0001	<0.0001	0.0002	<0.0001	<0.0001	<0.0001	<0.0001	<0.0001	<0.0001	<0.0001	<0.0001	<0.0001	<0.0001	<0.0001	<0.0001	<0.0001
Fish species	<0.0001	<0.0001	<0.0001	<0.0001	<0.0001	<0.0001	<0.0001	<0.0001	<0.0001	<0.0001	<0.0001	<0.0001	0.0037	<0.0001	0.0004	<0.0001
Fish species × Wood species	<0.0001	0.0121	0.0019	<0.0001	<0.0001	<0.0001	<0.0001	<0.0001	<0.0001	<0.0001	<0.0001	<0.0001	0.0022	<0.0001	<0.0001	<0.0001

Note: different superscripts show significant differences for *p* < 0.0001; Naph—naphthalene; Ace—acenaphthene; Flu—fluorene; Phen—phenanthrene; Ant—anthracene; Flt—fluoranthene; Pyr—pyrene; B(a)A—benzo(a)anthracene; Cry—chrysene; B(b)F—benzo(b) fluoranthene; B(k)F—ben-zo(k)fluoranthene; B(a)P—benzo(a)pyrene; D(a,h)A—dibenzo(a,h)anthracene; B(ghi)P—benzo(gy)pyrylene; I(1,2,3-cd)P—in-deno(1,2,3-cd)pyrene.

## Data Availability

The original contributions presented in the study are included in the article, further inquiries can be directed to the corresponding author.
